# Association between Oral Health-Related Quality of Life in People with Rare Diseases and Their Satisfaction with Dental Care in the Health System of the Federal Republic of Germany

**DOI:** 10.3390/ijerph15081732

**Published:** 2018-08-13

**Authors:** Marcel Hanisch, Sabrina Wiemann, Lauren Bohner, Johannes Kleinheinz, Susanne Jung

**Affiliations:** Department of Cranio-Maxillofacial Surgery, Research Unit Rare Diseases with Orofacial, Manifestations (RDOM), University Hospital Münster, Albert-Schweitzer-Campus 1, Building W 30, D-48149 Münster, Germany; s_wiem07@uni-muenster.de (S.W.); lauren@usp.br (L.B.); johannes.kleinheinz@ukmuenster.de (J.K.); susanne.jung@ukmuenster.de (S.J.)

**Keywords:** OHIP-14, oral health-related quality of life, OHRQoL, NAMSE, rare diseases

## Abstract

Background: The aim of this study was to examine the current dental care situation in Germany from the perspective of those affected by a rare disease, especially concerning their satisfaction with the German dental health care system, and thus assess the relationship between their perspective and their oral health-related quality of life (OHRQoL). Methods: A questionnaire regarding their experiences with the dental assistance and the health care system, such as the OHIP-14, was sent to the member associations of the organization of self-help groups for rare diseases ACHSE e.V. The correlation between OHIP-14 values and patient’s perspective was statistically analyzed by the non-parametric Tau de Kendall test (*p* < 0.05). Results: There was a statistically significant correlation between the OHIP score and the patient’s perspective regarding dental assistance and health care system (*p* < 0.05). For those surveyed who were satisfied with the support of the health care system, an average OHIP score of 8.54 ± 10.45 points (range: 0–48) was determined. The group that did not feel sufficiently supported by the health care system had an average OHIP score of 16.07 ± 13.43 points (range: 0–56). Discussion: The majority of respondents with rare diseases are dissatisfied with the German health care system and its support with regard to dental care.

## 1. Introduction

The definition of “rare diseases” changes worldwide. Whereas in the USA a disease is considered rare when fewer than 7.5 out of 10,000 people are affected by it, in Japan the disease is only defined as rare when it affects fewer than four out of every 10,000 people [1]. For Germany and the European Union, in turn, a disease is considered “rare” whether it affects fewer than five in 10,000 people [2]. This definition is estimated based on the 27–36 million people who are affected by a rare disease in the European Union, of which 4 million live in Germany [3]. In sum, around 8000 rare diseases are currently known, from which 15% can present oral manifestations [4,5].

Since 2009, rare diseases are increasingly gaining social relevance due to the commitment of European Union member states to establish and implement plans and strategies for rare diseases at an appropriate level to ensure adequate medical care for affected patients [2]. Thus, a National Action Plan for People with Rare Diseases was developed by the German Federal Ministry of Health in 2013 and it is currently in implementation [6]. This National Action Plan for People with Rare Diseases aims to improve the medical treatment situation of patients with rare diseases. Furthermore, it also supports health services research [6].

The Oral Health Impact Profile 14 (OHIP-14) questionnaire has proven itself to be a well-established method in order to assess the relation between oral health and quality of life [7], showing the impact of oral conditions on general health from a patient’s perspective [8]. Although few studies have assessed this outcome from the perspective of people with rare diseases [9], the existing literature suggests this population presents a reduced oral health-related quality of life [9,10,11,12]. In this respect, oral manifestations of rare diseases may be negatively associated with their oral health perception [12].

Furthermore, according to the authors’ knowledge, the satisfaction of those patients regarding their dental care and their satisfaction with the German dental health care system is still unknown. However, PricewaterhouseCoopers [13] showed that 79% of population were satisfied with health insurance benefits in the German healthcare system. Considering the special conditions required for people with rare diseases, there is a need to assess whether the dental care in the German health care system satisfy their needs. Thus, the aim of this study was to examine the current dental care situation in Germany from the perspective of those affected by a rare disease, especially concerning their satisfaction with the German dental health care system, and thus assessing the relation between their standpoint and the oral health-related quality of life.

## 2. Methods

### 2.1. Study Design

The present research has been approved by the Ethics Committee of the Ärztekammer Westfalen-Lippe and the Westfälische Wilhelms Universität Münster (Ref. No. 2016-006-f-S). The study was designed as an anonymous, epidemiological survey among people with rare diseases to evaluate their satisfaction with dental care in the healthcare system of the Federal Republic of Germany as well as their respective oral health-related quality of life.

For this purpose, a survey questionnaire composed of free text questions and the standardized German version of the OHIP-14 (Oral Health Impact Profile) was developed. The free text questions addressed the perception of the participants regarding their satisfaction with the dental treatment and the health care system in Germany.

The OHIP-14 questionnaire is the validated short form of the OHIP-49 and gathers information on the frequency of functional limitations, pain, psychological unease/discomfort, physical impairment, mental impairment, social impairment and disadvantage/disability in the past month [14] (Table S1). The responses are recorded on a Likert scale, of which the maximum number of points (56) suggests a very high impact on the oral health-related quality of life of the patient [7].

The snowball method was chosen to perform the distribution of questionnaires due to the difficulty of reaching people with rare diseases. The questionnaire in electronic file format was sent digitally to all 125 German member associations of the umbrella organization of self-help groups, the Alliance of Chronic Rare Diseases (ACHSE e.V.) in February 2016. All questionnaires received until July 2017 were considered.

### 2.2. Participants

All people affected by a rare disease in the Federal Republic of Germany from the age of 16 were eligible to take part of this study. Since the questionnaires were sent to the member associations of the ACHSE e.V., the participants were largely limited to the associated members.

### 2.3. Data Source

Descriptive data (age, gender and illness) were recorded and details regarding the dental treatment were assessed using dichotomous questions (Yes/No) concerning the difficulties of finding a dentist to perform the dental treatment, regardless of the rare disease. In addition, the satisfaction with the dentist and with the German health care system were recorded (Satisfied/Dissatisfied), as well as the travel time required to arrive at the dental clinic or hospital. Moreover, further questions addressed the patient’s perception towards the support provided by the German dental health care system

The OHIP-14 questionnaire consists of surveys regarding the limitations, pain, discomfort and disability experienced by the patient on the last month. The answers were assigned with a Likert scale for each of the 14 questions: 0 = never, 1 = rarely, 2 = from time to time, 3 = often and 4 = very often. Overall, a total value of 0 to a maximum of 56 points can be achieved. The higher the numerical value, the worse the oral health-related quality of life.

### 2.4. Statistical Analysis

Statistical analysis was performed using the SPSS 22.0 software (IBM). Data were described as percentage values, mean ± standard deviation, median (interquartile range) and 95% confidence interval (95% CI). The OHIP scores were calculated for different groups: “Gender”, “Difficulties in finding a dentist”, “Satisfaction with the dentist” and “Satisfaction with the health care system”.

Chi-square test (X^2^) was used to evaluate a possible association between dichotomic data. Further, the Kolmogorov–Smirnov test was performed to evaluate the adherence of data to the normal curve. The correlation between the OHIP values and the patient’s perspective were statistically analyzed using Tau de Kendall test with a significance level at *p* = 0.05.

## 3. Results

### 3.1. Participants

Considering that the questionnaire was distributed using the snowball method, it is not possible to determine how many people were reached. However, altogether 484 questionnaires were gathered from participants above 16 years old, from which 313 (64.66%) were women and 171 (35.33%) were men with 96 different rare diseases. The mean age (standard deviation) was 44.57 ± 16.40 years old.

### 3.2. Descriptive Data

Data concerning the travel time required to arrive at the dental clinic or hospital was converted in minutes. Within this purpose, information from 451 questionnaires were evaluated. The average travel time was therefore 22.01 ± 24.88 min. The longest travel time was reported as 360 min, whereas the lowest was zero minutes.

Respecting the limitations of the dental treatment, data were gathered from a total of 448 participants. In this regard, 92 (20.53%) participants argued having difficulties in finding a dentist who performed dental treatment regardless of their rare disease, whereas 356 (79.47%) participants had no difficulty in finding a dentist. The question regarding the satisfaction with their dentist was answered by 459 participants, of which 425 (92.59%) were satisfied and 34 (7.41%) were dissatisfied with their dentist.

The satisfaction of participants with the dental care in the German health system was gathered from 435 participants. According to this study, 190 people with rare diseases (43.68%) feel sufficiently supported by the German health care system, whereas 245 respondents (56.32%) answer negatively with regard to adequate support.

**Figure 1:**
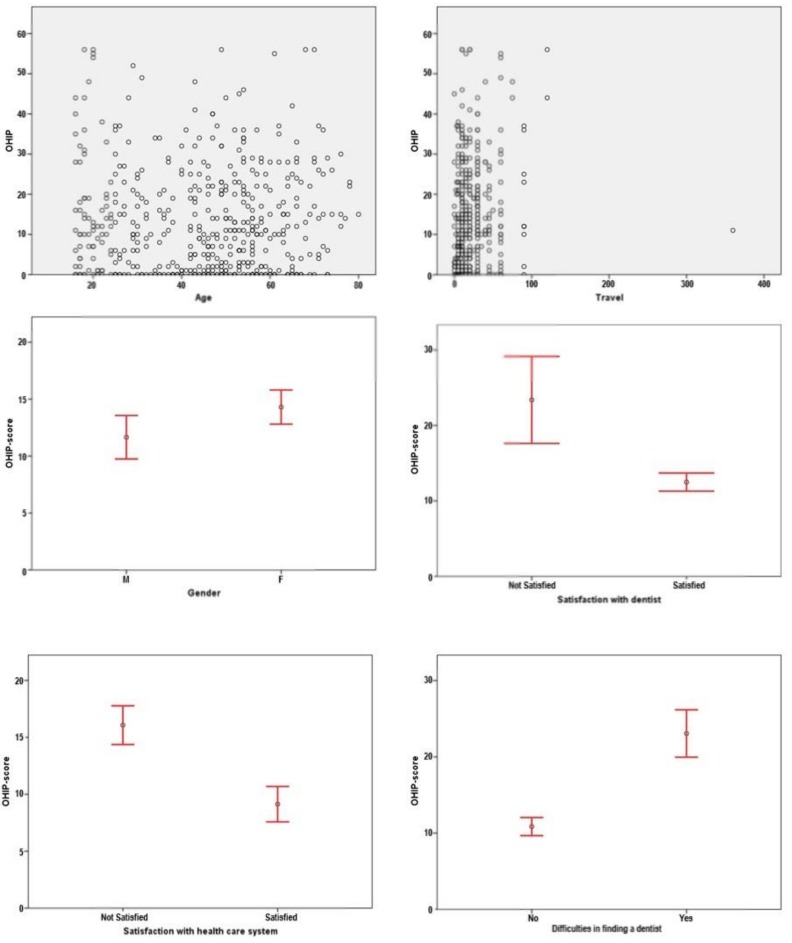
OHIP score according to different variables.

OHIP scores are described as median (Interquartile range) and graphically represented in Figure 1. According to the gender, OHIP-values were 9.00 (1–18) for male participants and 11.00 (2–22.25) for female participants. When evaluating the OHIP score considering their difficulties in finding a dentist, for those who had some difficulty the OHIP score was 22 (12–32), whereas for those who did not find any difficulty the OHIP score was 8.50 (0–17). Regarding the patient satisfaction with the dentist, the OHIP score was 10.00 (1–19) for the patients who were satisfied, and 21 (11–33) for those who were dissatisfied. Concerning the group who felt sufficiently supported by the German health care system, an OHIP score of 5.00 (0–15) was determined. The group who does not feel sufficiently supported by the German health care system had an OHIP score of 14.00 (5.50–25). Table 1 summarizes the commonly presented rare diseases (*n* ≥ 5), their OHIP-14 mean values, satisfaction with dentist and with the health system.

### 3.3. Statistical Analysis

There was no statistically significant association between the patient’s gender and their satisfaction with the dentist or with the health care system. Nonetheless, the number of patients satisfied or dissatisfied with the dentist was associated with their satisfaction regarding the health care system and to their difficulties in finding a dentist (Table 2).

The correlation between OHIP scores and patient’s data are described in Table 3. There was no correlation between the age and gender of participants and the OHIP scores, whereas a weak positive significant correlation (*p* < 0.05) was found for the travel time required to arrive at the dental clinic or hospital. That means, the higher the travel time that the participant requires, the higher will be the OHIP score. Additionally, participants who did show difficulties in finding a dentist tended to report greater OHIP scores (*p* < 0.05). The same significant correlation is assumed for those who reported to be dissatisfied with the dentist or with the health care system (*p* < 0.05)

## 4. Discussion

According to the authors’ knowledge, there are no studies assessing whether patients with rare diseases are satisfied with the dental care provided by the German health care system. Therefore, the aim of this study was to evaluate the current dental care situation in Germany from the perspective of those affected by a rare disease, especially concerning their satisfaction with the German dental health care system, and thus assessing the relationship between patient perspective and oral health-related quality of life.

### 4.1. Limitations

The questionnaire developed for the present study was limited to assessing patients’ perceptions regarding the dental assistance provided by the health care system. Nonetheless, the reasons why the patient felt satisfied or not were not investigated, and further studies are purposed to complement these data. Furthermore, 96 different rare diseases were analyzed in this study, and some of them showed a higher prevalence in comparison to others. Those predominant groups have more effect on the outcome, for instance, 32 participants with Ehlers–Danlos syndrome showed an OHIP mean value of 18.90). However, the aim of this study, founded on the political call for improving patient-centered-care, was to determine the relation between oral health-related quality of life and satisfaction with dental care in the health system of the Federal Republic of Germany not only for one patient group, but rather for all patients taking part in this research study.

### 4.2. Interpretation

People with rare diseases showed an OHIP score higher than the German general population [8,12]. Possibly, their oral health quality is lower due to limitations related to the disease, and it might get even worse in the absence of proper assistance.

The National Action Plan for People with Rare Diseases [6] in Germany is intended to set a three-stepped center structure for patients with rare diseases. In order to achieve this goal three types of centers must be established: A Center (reference center), B Center (expert center) and C Center (cooperation center). All of those centers are connected and cooperate with each other [6], and thus patients are able to conduct their treatment close to their homes in C Centers, which cooperate transregionally with A and B Centers. According to this study, the average amount of time a respondent needed to get to their dentist was 22.01 min. Study participants who experience a long drive to the nearest dental clinic potentially show higher OHIP values, which suggests a reduced oral health-related quality of life. Hence, it is possible that those patients may need an expert center due to their rare disease or that the nearest dentist does not have experience in treating rare diseases. 

This evidence leads to the conclusion that the framework of a center model, as provided for in the National Action Plan for People with Rare Diseases [15], comprising the cooperation of A or B Centers with community-based providers (C Centers), should be sought in order to provide home care to those affected. Respecting this, primary care centers (C Centers) should have the opportunity to cooperate and exchange experience with A and B Centers as a means to treat the patient optimally. This can be optimized through, e.g., further education events.

Additionally, approximately 20% of respondents reported difficulties in finding a dentist. Rare diseases are characterized in part by high complexity [16] and thus dentists may refuse to perform medical treatment for complex diseases or diseases that, like the Ehlers–Danlos syndrome or ectodermal dysplasia, can be risky or highly complex in the context of dental surgery or prosthodontics [17,18]. Many physicians/dentists do not have proper knowledge and experience in treating rare diseases [15], and this may explain why every fifth participant in the study revealed having difficulty in finding a dentist to perform dental treatment. This also reflects the imminent need for cooperation between primary centers and nationwide centers, in order to provide patients home care.

Overall, a high satisfaction with dental care is reported in the Federal Republic of Germany [19]. Predominantly, people with rare diseases who were interviewed in the present study reported a high level of satisfaction with their dentist, whereas only 7.41% were “dissatisfied” with their dentist. Concerning these patients, an average OHIP score approximately 2-times greater was shown in comparison with those satisfied with their dental assistance. It can therefore be assumed that the treatment quality seems to play an important role on the perception of patients regarding their oral health quality. However, it cannot be inferred that oral health-related quality of life is better because patients are satisfied with their dentist.

This study allowed us to receive more information regarding the oral health-related quality of life from the most prevalent rare diseases. For instance, patients with ectodermal dysplasia, on which hypodontia/oligodontia or anodontia are common manifestations, reported a reduced oral health-related quality of life. In this study, patients with ectodermal dysplasia showed high OHIP values (15.32) and only 15.56% were satisfied with the German health care system. In this respect, this could be improved by the offer of rehabilitation treatments, such as implant supported dentures [18]. Likewise, the reduced oral health-related quality of life compared with the average population, which is also described by other authors, can be confirmed for Ehlers–Danlos syndrome patients (18.90) [10]. Participants with Ehlers–Danlos syndrome were also mostly unsatisfied with the German healthcare system (62.97%). The disease is usually related with oral symptoms (e.g., hypodontia; temporomandibular dysfunction or periodontitis) [10,12,18]. The difficulty of accessing preventive and rehabilitation treatments, such as prophylaxis and implant supported dentures, may be the reason these patients are unsatisfied with the health care system.

Most participants of this study (56.32%) are “dissatisfied” with the support of the German health care system with regard to dental care. Respecting this, a recent study conducted by PricewaterhouseCoopers [13] revealed that 79% of respondents were satisfied with health insurance benefits in the German healthcare system. Although the study by PricewaterhouseCoopers has not explicitly analyzed satisfaction with dental services in the German health care system, it can be inferred from the data available that people with rare diseases are significantly less satisfied with the German health care system than the general population.

The association between oral health-related quality of life and satisfaction with the health care system is statistically significant. While participants who are satisfied with the health care system averaged an OHIP score of 8.54 ± 10.45 points, the score for dissatisfied participants is almost twice as high at 16.07 ± 13.43 points. This means that people with rare diseases who are satisfied with the health care system present a statistically and significantly better OHIP score than when they are dissatisfied. However, it cannot be assumed that the oral health-related quality of life is better because the participants are satisfied with the health care system. On the basis of this data it was not possible to find out why patients with rare diseases are not content with the German health care system. Future studies should address this question further. However, this may be an indication that additional support for dental care may be needed with regard to rare diseases.

An interesting observation is that, although most of the participants are satisfied with their dentists, they reported a negative perception from the German health care system in terms of dental assistance. This may be due to the bureaucratic requirements from the health care system, for instance, to provide free access to preventive or rehabilitation treatments to complications related to rare diseases such as x-linked hypophosphatemia, which shows higher risk for pulp necrosis and abscesses on caries-free teeth due to a modification of hard tissues [20]. From these patients, 66.93% were unsatisfied with the healthcare system.

## 5. Conclusions

People surveyed with rare diseases showed an OHIP score lower than the general German population. More than 90% of the study participants are “satisfied” with their dentist. The majority of respondents with rare diseases, on the other hand, are dissatisfied with the German health care system and its support with regard to dental care. A center model providing dental assistance close to home care with a cooperation between reference and primary centers might provide an improvement of patient care. Subsequently, it could also lead to an improvement of the oral health-related quality of life. Bureaucratic obstacles, oral symptoms and complicated access to important individual dental treatments like prophylaxis or implant supported dentures could also be the reason these patients are unsatisfied with the health care system.

## Figures and Tables

**Table 1:** Oral health status and satisfaction with German dental care in rare diseases. OMIM = Online Mendelian Inheritance in Man (available at www.omim.org).

Name of Disease	OMIM Number	Number of Individuals	Mean OHIP Value	Satisfied with Dentist	Satisfied with Health System
Marfan syndrome	154700	51	13.10	90.00%	44.44%
Ectodermal dysplasia	305100	46	15.32	97.61%	15.56%
Achalasia	231550	44	8.72	95.12%	51.28%
Sarcoidosis	181000	36	13.11	97.61%	51.63%
Ehlers–Danlos syndrome	120180	32	18.90	76.77%	27.03%
Lymphangioleiomyomatosis	606690	17	6.35	100%	53.33%
Alpha-1-Antitrypsin deficiency	613490	15	5.40	93.33%	60.00%
Syringomyelia	118420	13	20.91	90.91%	14.29%
X-linked hypophosphatemia	307800	13	13.85	83.33%	23.07%
Fabry disease	301500	8	7.38	100%	42.86%
Tuberous sclerosis	191100 613254	7	12.86	100%	16.67%
Von Willebrand disease	193400 277480 314560 613554	7	6.00	100%	100%
Lupus erythematosus	614420	6	15.17	100%	100%
Spinocerebellar ataxia	164400	6	11.33	100%	66.67%
Poliomyelitis	N/A	5	4.00	100%	60.00%
Prader–Willi syndrome	176270	5	6.60	60.00%	60.00%
Systemic sclerosis	181750	5	36.00	100%	25.00%

**Table 2:** Chi-square test.

	**Satisfaction with the Dentist**				
**Sex**	**Dissatisfied**	**Satisfied**	**Total**	**X^2^-Test**	***p*-Value**
**Male**	10	145	155	0.312	0.577
**Female**	24	280	304		
**Total**	34	425	459		
	**Satisfaction with the public system**				
**Sex**	**Dissatisfied**	**Satisfied**	**Total**	**X^2^-Test**	***p*-Value**
**Male**	93	67	160	0.335	0.563
**Female**	152	123	275		
**Total**	245	190	435		
**Satisfaction with the public system**	**Satisfaction with the dentist**				
	**Dissatisfied**	**Satisfied**	**Total**	**X^2^-Test**	***p*-Value**
**Dissatisfied**	24	201	225	5.818	0.016
**Satisfied**	8	179	187		
**Total**	32	380	412		
**Difficulties in finding a dentist**	**Satisfaction with the dentist**				
	**Dissatisfied**	**Satisfied**	**Total**	**X^2^-Test**	***p*-Value**
**No**	22	325	347	4.838	0.028
**Yes**	11	70	81		
**Total**	33	395	428		

**Table 3:** Correlation between OHIP values and the patient’s perspective. *n* = sample size, τ = Kendall’s Tau. OHIP values are shown as mean ± standard deviation.

	*n*	OHIP Values	τ	*p*-Value
Age	480		0.009	0.392
Travel time	480		0.132 *	0.000
Sex	480	Male: 11.60 ± 12.10	0.081 *	0.018
		Female: 13.59 ± 12.91		
Difficulties in finding dentist	448	Yes: 23.05 ± 15.01	0.293	0.00
		No: 10.86 ± 11.28		
Satisfaction with Dentist	452	Dissatisfied: 23.38 ± 16.52	−0.157 *	0.000
		Satisfied: 12.56 ± 12.45		
Satisfaction with the System	428	Dissatisfied:16.07 ± 13.43	−0.242 *	0.000
		Satisfied: 8.54 ± 10.45		

* Statistical significance (*p* < 0.05).

## Supplementary Materials

The following are available online at http://www.mdpi.com/1660-4601/15/8/1732/s1, Table S1: original version of the questionnaire.
