# Supplementary material for: Association between Oral Health-Related Quality of Life in People with Rare Diseases and Their Satisfaction with Dental Care in the Health System of the Federal Republic of Germany
Source: Int J Environ Res Public Health. 2018 Aug 13;15(8):1732. doi: 10.3390/ijerph15081732 (PMC6121257; doi:10.3390/ijerph15081732)
Supplement: Supplementary file 1 [file ijerph-15-01732-s001.pdf]

Supplementary Material

Table S1. Original version of the questionnaire.

**Hatten Sie <u>im vergangenen Monat</u> aufgrund von Problemen mit Ihren Zähnen, im Mundbereich oder mit Ihrem Zahnersatz …**	**sehr oft**	**oft**	**ab und zu**	**kaum**	**nie**
….Schwierigkeiten bestimmte Worte auszusprechen?					
….das Gefühl, Ihr Geschmackssinn war beeinträchtigt?					
….den Eindruck, dass Ihr Leben ganz allgemein weniger zufriedenstellend war?					
….Schwierigkeiten zu entspannen?					
**Ist es <u>im vergangenen Monat</u> aufgrund von Problemen mit Ihren Zähnen, im Mundbereich oder mit Ihrem Zahnersatz vorgekommen, …**	**sehr oft**	**oft**	**ab und zu**	**kaum**	**nie**
….dass Sie sich angespannt gefühlt haben?					
….dass Sie Ihre Mahlzeiten unterbrechen mussten?					
….dass es Ihnen unangenehm war, bestimmte Nahrungsmittel zu essen?					
….dass Sie anderen Menschen gegenüber eher reizbar gewesen sind?					
….dass es Ihnen schwergefallen ist, Ihren alltäglichen Beschäftigungen nachzugehen?					
….dass Sie vollkommen unfähig waren, etwas zu tun?					
….dass Sie sich ein wenig verlegen gefühlt haben?					
….dass Ihre Ernährung unbefriedigend gewesen ist?					
**Hatten Sie <u>im vergangenen Monat</u> …**	**sehr oft**	**oft**	**ab und zu**	**kaum**	**nie**
….Schmerzen im Mundbereich?					
….ein Gefühl der Unsicherheit in Zusammenhang mit Ihren Zähnen, Ihrem Mund oder Ihrem Zahnersatz?					

OHIP-G 14 * John M, Micheelis W, Biffar R. Einflussfaktoren mundgesundheitsbezogener Lebensqualität - Validierung einer deutschen Kurzversion des Oral Health Impact Profile (OHIP-G 14). *Dtsch Zahnärztl Z.* **2004**, *59*, 328–333.
